# A 26-hour system of highly sensitive whole genome sequencing for emergency management of genetic diseases

**DOI:** 10.1186/s13073-015-0221-8

**Published:** 2015-09-30

**Authors:** Neil A. Miller, Emily G. Farrow, Margaret Gibson, Laurel K. Willig, Greyson Twist, Byunggil Yoo, Tyler Marrs, Shane Corder, Lisa Krivohlavek, Adam Walter, Josh E. Petrikin, Carol J. Saunders, Isabelle Thiffault, Sarah E. Soden, Laurie D. Smith, Darrell L. Dinwiddie, Suzanne Herd, Julie A. Cakici, Severine Catreux, Mike Ruehle, Stephen F. Kingsmore

**Affiliations:** Center for Pediatric Genomic Medicine, Children’s Mercy, 2401 Gilham Road, Kansas City, MO 64108 USA; Department of Pediatrics, Children’s Mercy, Kansas City, MO 64108 USA; Department of Pathology, Children’s Mercy, Kansas City, MO 64108 USA; School of Medicine, University of Missouri-Kansas City, Kansas City, MO 64108 USA; Deparment of Pediatrics, and Clinical Translational Science Center, University of New Mexico Health Science Center, Albuquerque, NM 87131 USA; Edico Genome, Inc., 3344 North Torrey Pines Court, Plaza Level, La Jolla, CA 92037 USA; Rady Pediatric Genomics and Systems Medicine Institute, Rady Chlildren’s Hospital, 3020 Children’s Way, San Diego, CA 92123 USA

## Abstract

While the cost of whole genome sequencing (WGS) is approaching the realm of routine medical tests, it remains too tardy to help guide the management of many acute medical conditions. Rapid WGS is imperative in light of growing evidence of its utility in acute care, such as in diagnosis of genetic diseases in very ill infants, and genotype-guided choice of chemotherapy at cancer relapse. In such situations, delayed, empiric, or phenotype-based clinical decisions may meet with substantial morbidity or mortality. We previously described a rapid WGS method, STATseq, with a sensitivity of >96 % for nucleotide variants that allowed a provisional diagnosis of a genetic disease in 50 h. Here improvements in sequencing run time, read alignment, and variant calling are described that enable 26-h time to provisional molecular diagnosis with >99.5 % sensitivity and specificity of genotypes. STATseq appears to be an appropriate strategy for acutely ill patients with potentially actionable genetic diseases.

**Electronic supplementary material:**

The online version of this article (doi:10.1186/s13073-015-0221-8) contains supplementary material, which is available to authorized users.

## Background

Genomic medicine is a new discipline whereby an individual’s genome information is used to guide personal strategies for disease prevention, etiologic diagnosis, and therapeutic selection [1, 2]. Despite its recent implementation into clinical care, genomic medicine is already transforming the diagnosis, molecular staging, prognostic determination, and management of patients with symptoms suggestive of genetic diseases, particularly Mendelian disorders (those caused by defects in single genes) and recurrent cancers [3–11]. Genomic medicine is transformative in these applications because it rapidly and simultaneously tests nearly all genes potentially relevant to that patient’s disease, largely irrespective of the clinician’s differential diagnosis list or detailed knowledge of all of the conditions being tested [6, 11]. This is particularly powerful for patients with very rare or newly discovered diseases, atypical clinical presentations or responses to treatment, and actionable pharmacogenomic findings. Timely molecular diagnosis, staging, and prognosis along with pharmacogenomic-based guidance can immediately engender a treatment shift from interim, phenotype-driven, population-based management to precision medicine with definitive, individualized therapies, and management plans, as well as drug exposures attuned to genotype and molecular prognosis [2]. In particular, there is increasing evidence that rapid whole genome sequencing (WGS) can be useful in the acute care of infants with genetic diseases in neonatal and pediatric intensive care units [6, 11–13].

While the cost of WGS has fallen dramatically, it remains too slow to be suitable for guidance in the management of many acute medical conditions. We previously described diagnostic WGS for genetic diseases in 50 h (WGS_50_), with 77–96 % sensitivity and approximately 99.5 % specificity for detection of nucleotide variants [12]. Fifty hours was the interval between receipt of a blood sample and identification of a provisional molecular diagnosis, provided that the diagnosis was readily apparent upon variant filtering. WGS_50_ had two principal time components: 2 × 100 cycles of sequencing-by-synthesis (SBS) was approximately 25.5 h. The identification of nucleotide variants by sequence alignment, variant calling, and genotyping was approximately 17.5 h. Here we describe second generation STATseq, with improved timeliness, sensitivity, and scalability.

## Methods

### Study design, setting, and participants

This study was approved by the Institutional Review Board (IRB) at Children’s Mercy – Kansas City (CM-KC). It conforms to the Declaration of Helsinki. Participants were principally parent–child trios enrolled in a research biorepository who received WGS in addition to standard diagnostic tests to diagnose monogenic disorders of unknown etiology in affected infants [6, 11, 12]. Affected infants with suspected genetic disorders were nominated by a treating physician, typically a neonatologist. A standard form requesting the primary signs and symptoms, past diagnostic testing results, differential diagnosis or candidate genes, pertinent family history, availability of biologic parents for enrollment, and whether rapid WGS would potentially affect treatment was submitted for immediate evaluation by a team of experts at the Center for Pediatric Genomic Medicine (CPGM) at CM-KC. Infants received rapid WGS if the likely diagnosis was of a type that was detectable by next-generation sequencing and had any potential to alter management or genetic counseling. Patients were not required to undergo standardized clinical examinations or diagnostic testing prior to referral; standard etiologic testing was performed as clinically indicated. Infants likely to have disorders associated with cytogenetic abnormalities were not accepted unless standard testing for those disorders was negative. Informed written consent was obtained from parents. Retrospective samples, UDT103 and UDT173, were blinded validation samples with known molecular diagnoses for a genetic disease [12]. Reference sample NA12878 was obtained from the Coriell Institute repository.

### Ascertainment of clinical features

The clinical features of affected infants were ascertained comprehensively by physician and family interviews and review of the medical record. Phenotypic features were translated into Human Phenotype Ontology (HPO) terms and mapped to 6,000 genetic diseases with the clinicopathologic correlation tools Phenomizer and/or SSAGA [6, 11, 14, 15]. Briefly, Phenomizer uses term-similarity measures to calculate a similarity score for query HPO terms entered by the user and terms used to annotate diseases in HPO. It then assigns a *P* value using statistical modeling to compare the similarity score obtained for the specific set of phenotypic terms entered into the distribution of similarity scores obtained using randomly chosen HPO term combinations. The *P* value was then used to rank diseases in the differential genetic diagnosis. The Phenomizer differential genetic diagnosis is exported as a tab-separated value file. Diagnoses without known causative genes are removed. Where the likely inheritance pattern is apparent, the Phenomizer output is limited to the appropriate inheritance mode. Where Phenomizer reports many equally scoring values, the differential diagnosis is performed using several different but overlapping term sets, for example with a key feature being listed as mandatory rather than observed, or with removal of clinical features that are felt to likely represent a second, unrelated disorder. Finally, the Phenomizer list may be pruned to 100–250 entries, if necessary, based on manual inspection of the fit of diseases to the clinical features at various *P* value cutoffs.

Similarly, SSAGA is web-based software that facilitates entry of Human Phenotype Ontology (HPO) terms related to the clinical features observed in an individual patient (Additional file 1: Figure S1). SSAGA provides a differential diagnosis that is limited to all Online Mendelian Inheritance in Man (OMIM), Orphanet, and DECIPHER (DatabasE of genomiC varIation and Phenotype in Humans using Ensembl Resources) disease entries that match at least one entered feature [16, 17]. Diseases in the differential genetic diagnosis can be ranked by the number of matching terms entered.

### Genome sequencing and quality control

DNA isolation from peripheral blood was automated utilizing the MSMI Chemagen Instrument equipped with liquid dispensing manifolds (Perkin Elmer, Baesweiler, Germany). Briefly, a 24-well head is used to isolate 1.8 mL of blood per sample. The system is fully enclosed to comply with biosafety standards, and isolation is completed in approximately 2 h resulting in an average of approximately 40 μg of DNA/mL of blood.

For 18-h WGS performed in Essex, isolated genomic DNA was prepared using a modification of the standard Illumina TruSeq sample preparation. Briefly, DNA was sheared using a Covaris S2 Biodisruptor, end-repaired, A-tailed, and adaptor-ligated. PCR was omitted. Libraries were purified using SPRI beads (Beckman Coulter). For 18-h WGS, the amount of DNA used was optimized, based on experience of varying the input from representative DNA samples, and allowed a concentration to be selected that produced a known cluster density after the library was denatured using 0.1 M NaOH and presented to the flowcell.

At CM-KC, genomic DNA was prepared for WGS using either TruSeq PCR Free (Illumina) or KAPA HYPER (KAPA Biosystems) without PCR amplification according to manufacturer’s protocols. HYPER library preparation without PCR is completed in 90 min, with an additional 90 min allotted for QC. Briefly, 2 μg of DNA was sheared with a Covaris S2 Biodisruptor, end-repaired, A-tailed, and adaptor-ligated. Quantitation of libraries was carried out by real-time PCR.

Samples for rapid WGS were each loaded onto one or two flowcells, followed by sequencing on Illumina HiSeq2500 instruments using HiSeq Rapid SBS v1 chemistry that were set to rapid run mode (SBS_26_) or with customized faster flowcell scanning times (SBS_18_). Cluster generation, followed by 2 × 101 cycle sequencing reads, separated by the paired-end turnaround, were performed automatically on the instrument. Prior to SBS_18_ optimization, tiles failing quality metrics were manually excluded before further analysis. Genome sequencing was performed as research, largely in a manner that complies with routine diagnostic tests, as defined by the Clinical Laboratory Improvements Act (CLIA) guidelines [18–21]. However, genome sequencing was not performed herein as a CLIA laboratory developed test.

### Next generation sequencing analysis

Sequence data were generated with Illumina RTA 1.12.4.2 & CASAVA-1.8.2, aligned to the human reference GRCh37.p5 using GSNAP [22], and nucleotide (nt) variants were detected and genotyped with the Genome Analysis Tool Kit [23] (GATK, versions 1.6. and 3.2). Sequence analysis used FASTQ, bam, and VCF files. The largest deletion variant detected was 263 nt, and the largest insertion was 469 nt.

Variants were annotated with the Rapid Understanding of Nucleotide variant Effect Software (RUNES, v3.3.5) [6, 11, 12]. RUNES incorporates data from ENSEMBL’s Variant Effect Predictor (VEP) software [24], produces comparisons to NCBI dbSNP, known disease variants from the Human Gene Mutation Database [25], and performs additional *in silico* prediction of variant consequences using RefSeq and ENSEMBL gene annotations [26]. RUNES categorized each variant according to ACMG recommendations for reporting sequence variation [18–21] and with an allele frequency (MAF) derived from CPGM’s Variant Warehouse database of approximately 90 million variants and 3,900 individuals [6, 11, 12]. Category 1 variants had previously been reported to be disease-causing. Category 2 variants had not previously been reported to be disease-causing, but were of types that were expected to be pathogenic (loss of initiation, premature stop codon, disruption of stop codon, whole gene deletion, frameshifting indel, disruption of splicing). Category 3 were variants of unknown significance that were potentially disease-causing (non-synonymous substitution, in-frame indel, disruption of polypyrimidine tract, overlap with 5’ exonic, 5’ flank or 3’ exonic splice contexts). Category 4 were variants that were probably not causative of disease (synonymous variants that were unlikely to produce a cryptic splice site, intronic variants >20 nt from the intron/exon boundary, and variants commonly observed in unaffected individuals). Category 5 variants were known to be benign. All variants, together with their RUNES annotations, are stored in a queriable warehouse database (Additional file 2: Figure S2). Inputs to the RUNES pipeline were a genomic variant file (.vcf or .gvcf); the pipeline produces a JSON document that is used as input to the VIKING interpretation tool.

### DRAGEN

The DRAGEN pipeline operates on a single-server hybrid hardware/software platform, with a dual Intel Xeon central processing units (CPUs), and a custom Peripheral Component Interconnect Express (PCIe) board with a field-programmable gate array (FPGA) and 32 GB of Dynamic random-access memory (DRAM) attached directly via four double data rate type three synchronous dynamic random-access memory DDR3 SDRAM channels. Critical compute-intensive functions of the pipeline are performed by custom massively parallel FPGA logic for maximum speed, while other functions run in optimized multi-threaded software on the Xeon cores, for maximum flexibility. A parallel (redundant array of independent disks, RAID 0) Solid State Drive (SSD) file system provides the I/O bandwidth necessary to feed the processing pipeline, and FPGA compress/decompress engines maintain throughput to and from compressed file formats.

### DRAGEN read mapping/alignment

DRAGEN uses a hash table index of a reference genome to map many overlapping seeds from each read to exact matches in the reference. The hash table is constructed from any chosen reference with a multi-threaded tool, in as little as 6 min for a whole human genome, and loaded into the FPGA-board DRAM prior to mapping operations. The entire read mapping process is performed by custom FPGA logic, with software layers streaming unaligned reads from FASTQ (or Illumina BCL) files to the PCIe board via DMA, and simultaneously streaming aligned read records back into host memory, for BAM output and/or variant calling.

DRAGEN’s hash-based mapping uses a novel dynamic seed extension method: when a primary seed (default 21 nt) matches more than a maximum number of reference locations (default 16), longer seeds from all these reference positions are populated into the hash table, such that specific extended seed sequences will match fewer reference locations. Seeds are extended symmetrically, up to 64 nt in each direction, with a maximum of 149 nt from a 21 nt primary seed. Long seed extensions were done in multiple short increments, averaging 3–4 nt in each direction, with different extended seed patterns terminating at different extension lengths, as needed to match no more than the maximum number of reference positions.

When a hash table query is made for a common primary seed, a single EXTEND record (merging the contents of two or more objects together into the first object) is retrieved, indicating the number of additional read bases to join onto the seed in each direction. The additional bases were hashed (along with a unique identifier for the pre-extended seed), and another hash table query was made, which may return yet another EXTEND record, iteratively. When an adequate extended seed length was achieved, the next hash table query retrieved a list of up to the maximum number of matching reference positions and orientations. This iterative seed extension method yields similar results to incremental suffix-tree or Burrows-Wheeler mapping but with dramatically fewer index memory accesses, which is critical to DRAGEN’s mapping speed.

In FPGA logic, read pairs are load-balanced over several DRAGEN map/align engines. An engine extracts many overlapping seeds from each read, by default starting at every even offset (50 % density). These are mapped by DRAM hash table queries, each to zero or more reference positions, with forward or reverse-complemented orientation determined for each match. The several engines nearly saturate the four local DDR3 interfaces with hash bucket read bursts and reference sequence fetches for alignment. Matches along similar alignment diagonals are grouped into seed chains, which are conservatively filtered; by default, a short seed chain can be filtered out if another seed chain at least four times longer mostly overlaps it in the read.

Lists of seed chains from paired end reads are examined to detect pairs with appropriate insert sizes and orientations. For each unpaired seed chain, a rescue scan may be executed to search for the mate within the expected insert window; mate K-mer matches within a configurable Hamming distance (the number of positions at which the corresponding nucleotides are different) result in new candidate positions being added to the list of seed chains. Each seed chain or rescue match is then extended by gapless local alignment, permitting single nucleotide variants (SNVs) and clipping but not nucleotide insertions and deletions (indels). The collection of gapless alignment results for each read is analyzed by heuristics, to judge for which ones Smith-Waterman gapped alignment would have a non-trivial likelihood of improving the overall read pair results.

Each Smith-Waterman aligner uses an array of 56 parallel scoring cells, virtually arranged into an anti-diagonal wavefront, which steps one position horizontally or vertically each clock cycle. The wavefront scores a generally diagonal swath of cells through the alignment rectangle but steers automatically to re-center the best alignment path after indel events. Back-trace from the maximum scoring cell runs simultaneous with the following alignment operation, yielding a CIGAR string, which indicates soft clipping and indel positions.

All gapped and gapless alignment results are compared to obtain best and second-best scores. For paired ends, pair scores are computed, each as the sum of the two alignment scores minus a pairing penalty, based on the deviation from the empirical mean insert; and the best scoring pair is reported. The quality of read mapping (MAPQ) is estimated primarily in proportion to the difference between best and second best scores, the proportionality coefficient varying by read length; second-order factors such as the number of scores very close to the second-best are also considered. When the best alignment does not cover a read, up to three supplementary (chimeric) alignments are optionally reported for other segments of the read.

### DRAGEN sorting and duplicate marking

After mapping, reads are sorted by reference position; PCR or optical duplicates are optionally flagged. An initial sorting phase operates on aligned reads returning from the FPGA. Final sorting and duplicate marking commences when mapping completes; these operations overlap variant calling when the latter is requested, and add almost zero time to the FASTQ-to-VCF pipeline.

### DRAGEN variant calling

The DRAGEN variant caller runs mostly in highly optimized software, for maximum flexibility of the algorithms. Only stable, compute-intensive operations are accelerated by FPGA engines. DRAGEN implements multi-threaded parallelism in a single pass over the whole reference genome, without launching multiple caller processes on various subsets of the reference. A single call to the DRAGEN executable runs the entire pipeline from FASTQ to VCF, for the whole genome. Mapping/alignment is done in one pass over the reads, and all steps of variant calling (in addition to read sorting and duplicate marking) run simultaneously in a software/hardware pipeline emitting VCF results.

First, callable reference regions are identified, with sufficiently aligned coverage. Within these, a fast scan of the sorted reads identifies active regions, centered around pileup columns with non-trivial evidence of a variant, and padded with enough context to cover significant non-reference content nearby, extra wide where there is evidence of indels.

Aligned reads are clipped within each active region and assembled into a De Bruijn graph, edges weighted by observation counts using the reference sequence as a backbone. If the graph is degenerate, it is reconstructed using longer K-mers. After some graph cleanup and simplification, all source-to-sink paths are extracted as candidate haplotypes, up to a limit (default 128). If this cap must be enforced, higher-weight paths are preferred. Each haplotype is Smith-Waterman aligned back to the reference genome to identify the variants it represents, and re-synchronized with read alignments.

Then for each read-haplotype pair, the probability P(r|H) of observing the read, assuming the haplotype was the true starting sample, is estimated using a pair hidden Markov model (HMM). Since the haplotype is assumed true, only errors in sample preparation and sequencing are considered. Essentially, the probabilities of all possible alignments (edit combinations) of the read to the haplotype are calculated and summed, using a dynamic programming matrix very similar to affine-gap Smith-Waterman, except summing rather than maximizing path probabilities. At each row (read position) in the matrix, mismatch probabilities are taken from base quality scores and MAPQ, and gap probabilities are derived from a PCR error model sensitive to repetitive sequence content.

This pair-HMM calculation is the most expensive step, and, therefore, is accelerated in custom FPGA logic. Reads and haplotypes to be compared are queued up for HMM processing by software threads completing previous steps and sent to the FPGA by direct memory access (DMA). A load balancer distributes work over more than 100 small HMM engines, each of which is pipelined to calculate all three probabilities (for match, insert, and delete states) for one matrix cell per clock cycle. Calculated P(r|H) values DMA back to host memory, where they are picked up by downstream software threads.

Scanning by reference position over the active region, candidate genotypes are formed from diploid combinations of variant events (SNVs or indels) observed in the earlier Smith-Waterman alignments of the haplotypes to the reference. For each event (including reference), the conditional probability P(r|e) of observing each overlapping read is estimated as the maximum P(r|H) for haplotypes supporting the event. These are multiplied to yield the conditional probability P(R|e) of observing the whole read pileup, and using Bayes’ formula, the posterior probability P(e1e2|R) of each diploid genotype (diplotype) is calculated, and the winner is called.

### VIKING

Causative variants were identified primarily with Variant Integration and Knowledge INterpretation in Genomes (VIKING) software (Additional file 2: Figure S2 and Additional file 3: Figure S3) [6, 11]. Inputs for VIKING were the annotated genomic variant file produced by the RUNES pipeline and a SSAGA (Symptom and Sign Associated Genome Analysis) or Phenomizer record, comprising the clinical features of the affected patient, corresponding diseases in the differential diagnosis, and the respective disease genes (Additional file 1: Figure S1) [6, 11, 14, 15]. The SSAGA or Phenomizer record was created during the laboratory steps in WGS_26_. Alternatively, a menu of pre-determined candidate gene lists can be utilized to filter variants in VIKING, such as genes with OMIM records, or genes previously associated with mitochondrial disorders. VIKING integrated the superset of relevant disease mappings and annotated variant genotypes. By allowing dynamic filtering of variants based on variables such as individual clinical features, diseases, genes, assigned ACMG-type pathogenicity category, allele frequency, genotype, and inheritance pattern, VIKING assists in identification of a differential diagnosis. VIKING settings can be saved, which allows configuration in a manner that can enable a provisional molecular diagnosis to be determined in as little as seconds. VIKING also allowed data mark-up, sessions to be saved, and export of fields in formats suitable for inclusion in diagnostic reports.

In a typical interpretation session, variants were filtered by limitation to ACMG Categories 1–3 and MAF <1 %, <0.5 %, <0.1 %, or to those that are unique to the proband or to the family, dependent on the clinical impression (Additional file 3: Figure S3). All potential monogenetic inheritance patterns were examined, including *de novo*, recessive, dominant, X-linked, mitochondrial, and, where possible, somatic variation. Where a single likely causative heterozygous variant for a recessive disorder was identified, the entire coding domain was manually inspected using the Integrated Genome Viewer (IGV) for coverage, additional variants, as were variants for that locus called in the appropriate parent that may have had low coverage in the proband [27]. VIKING featured link-outs to IGV that are refreshed in a trio on a variant-by-variant basis allowing rapid examination of pattern of inheritance, quality of alignment, and local sequence features (such as simple sequence repeats). Expert interpretation and literature curation were performed for all likely causative variants with regard to evidence for pathogenicity. VIKING featured link-outs to a warehouse of approximately 90 million variants in approximately 3,900 individuals, OMIM, HGNC, HGMD, Entrez Gene, ENSEMBL and pathways information, facilitating rapid literature curation (Additional file 2: Figure S2). Analysis was performed sequentially by two experts. Sanger sequencing was used for clinical confirmation of all diagnostic genotypes. Reporting was performed by an ACMG fellow laboratory director who was an expert in WGS analysis in single gene diseases. Additional expert consultation and functional confirmation were performed when the subject’s phenotype differed from previous mutation reports for that disease gene.

## Results

### 26-h whole genome sequencing

**Fig. 1:**
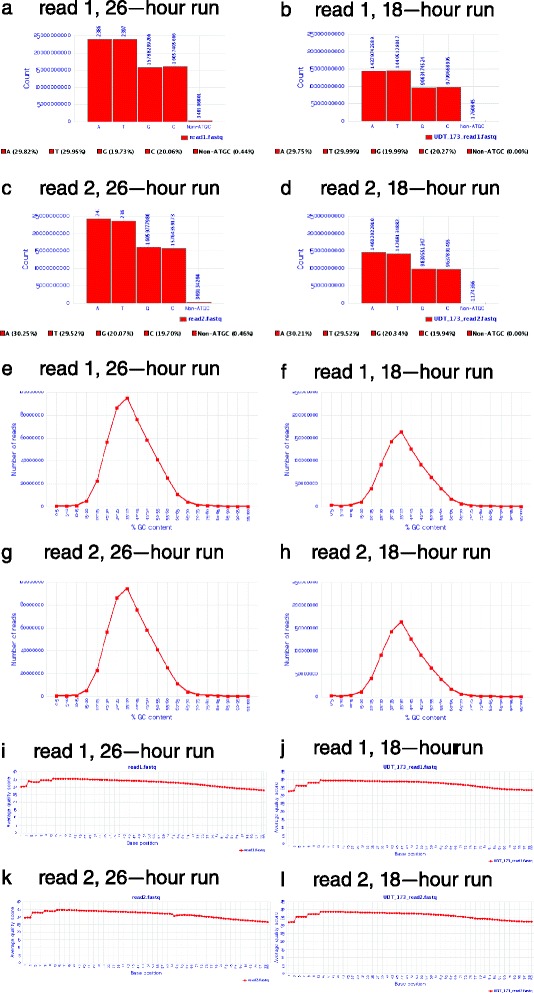
Comparison of quality metrics of 18-h and 26-h 2 × 100 nt runs. The runs were WGS of sample UDT_173 [12]. **a**–**d**. Base composition was not materially different in the 18-h and 26-h runs. However, the % non-AGTC reads was lower in the 18-h run. This may either reflect better sequence quality or lower cluster density. **e**–**h**. Frequency distribution of GC content of 18-h and 26-h runs. While the number of reads (y-axis) differed between runs, 18-h and 26-h runs had identical GC content distributions, with sequence representation between GC content of 15 % and 75 %. GC content varies widely across the human genome ― the isochore structure of the human genome [24, 35]. The median genome GC content estimated by 18-h and 26-h WGS (35–40 %) agreed with the estimated median from the 1,000 genomes project [36] (38.6 %), and is slightly lower than estimates by cesium density gradient centrifugation [42, 43] (39.6–40.3 %). **i**–**l**. Quality scores of nucleotide calls as a function of cycle were indistinguishable in 18-h and 26-h runs

WGS with a 26-h time from blood sample to provisional diagnosis (WGS_26_), was achieved by the acceleration of several components. First, 2 × 100 cycles of SBS, including on-board cluster generation, was reduced from 25.5 h to 18–21 h (SBS_18_). A total of 25.5 h was accomplished with the rapid run mode on the Illumina HiSeq 2500 sequencing instrument and 18–21 h was achieved by the development of an ultra-rapid run mode on the same instrument. In addition to recipe changes (faster cycles of sequencing by synthesis, SBS), this necessitated fine-tuning of ramping of heating and cooling during SBS, optimization of temperature uniformity across the flow cell, and adjustments to microfluidics. After optimization, the quality, quantity, and precision of sequences obtained with an 18–21-h run time was indistinguishable from that with 25.5-h runs (Table 1, Fig. 1). Cluster density on flow cells, not run time, was the major covariate for sequence yield, sequence quality, and error rate with an 18–21 h run time (Additional file 4: Table S1).

**Table 1:** Breakdown of times of principal steps for rapid diagnostic whole genome sequencing

Method	Sample	Site	DNA isolation, QC and shearing	PCR-free library prep	WGS library QC	SBS	Yield (GB)	% > Q30	Alignment	Variant calling	RUNES variant annotation	Provisional diagnosis	Total time
Published WGS_50_	Multiple^a^	Both	2:30	3:15	1:30	25:30	139	90	14:40		2:30	0:05	50:00
SBS_18_, GSNAP/GATK/noVQSR	5006-01	CMH	2:30	3:15	1:30	19:45	128	91	22:30		0:29	n.a.	49:59
WGS_26,_ SBS_18_, and Dragen v1.2	UDT_173	Essex	2:30	3:02	1:30	17:58	106	92	0:15	0:15	0:34	0:04	26:08
WGS_26,_ SBS_18_, and Dragen v1.2	UDT_103	Essex	2:30	3:05	1:30	18:25	130	90	0:19	0:22	0:31	0:05	26:47
WGS_26_, SBS_18_, and Dragen v1.2	NA12878	Essex	2:30	3:15	1:30	18:00	143	85^b^	0:19	0:22	0:33	n.a.	26:28
WGS_26_, SBS_18_, and Dragen v1.2	NA12878	CMH	2:30	3:15	1:30	18:36	65^c^	85^b^	0:10	0:11	0:35	n.a.	26:47

GB, gigabases; Q, Phred-like quality score QC, quality control; SBS, 2 × 101 cycle sequencing-by-synthesis

^a^Reference 12

^b^Prior to SBS_18_, after failing tiles were removed

^c^Single flowcell

Second, the time taken for sequence alignment, variant detection, and genotyping was reduced from approximately 15 h in WGS_50_, gapped alignment, and variant calling with CASAVA v1.8.2 (Illumina), to approximately 40 min for WGS_26_ with the novel DRAGEN aligner and variant caller (Table 1). DRAGEN accelerated these steps by highly parallel alignments to a sorted reference genome and customized high-memory computer hardware with high IO throughput.

Third, in WGS_50_ variants were annotated for likely functional consequence with Rapid Understanding of Nucleotide variant Effect Software (RUNES) software in 2.5 h. In WGS_26_, RUNES was accelerated to 30 min by software refactoring. Fourth, WGS_50_ utilized manual manipulation of spreadsheets for analysis and interpretation of variants. In WGS_26_, these steps were performed with the interpretation software VIKING (Variant Integration and Knowledge INterpretation In Genomes). VIKING facilitated genome analysis and interpretation by allowing dynamic filtering of variants based on variables such as individual clinical features, diseases, genes, assigned ACMG-type pathogenicity category, allele frequency, genotype, and inheritance pattern (Additional file 2: Figure S2). For example, VIKING filtering to display variants with: (1) an ACMG-type pathogenicity category of 1–3; (2) an allele frequency of less than 0.1 %; (3) that fit a recessive inheritance pattern (homozygous, compound heterozygous, or hemizygous); and (4) that are in OMIM monogenic disease-associated genes, yielded 16 variants in eight genes in WGS_18_ of sample UDT_103 (Additional file 2: Figure S2). Of these, only two variants in one gene fit the clinical features of the patient and a *bona fide* inheritance pattern. They were *UNC13D* NM_199242.2 c.2955-2A > G in 16 of 31 reads with quality score 99, which was inherited maternally, and c.859-3C > A in 18 of 35 reads with quality score 99, which was inherited paternally. These variants gave an actionable, provisional diagnosis of Hemophagocytic lymphohistiocytosis type 3. In complex cases, where no causative diplotype is identified by such semi-automated analysis, a thorough manual analysis ensues that can take many hours.

### Analytic performance of 26-h WGS

The analytic performance of WGS_26_ was examined at two sites (Illumina in Essex, Children’s Mercy Hospital, Kansas City, MO, USA), with three SBS_18_ sequencing instruments over a period of 2 years (Table 2). There was an evolution of sequencing instruments, software, computer hardware, and reference standards during this period. The alignment and variant calling algorithm of the original WGS_50_, CASAVA, had excellent specificity for detection of nucleotide variants (99.5 %), moderate sensitivity (77–86 %), and had a computation time of 14.5 h [12]. The alternate method described at that time was read alignment with GSNAP and variant detection with GATK and best practices, providing a sensitivity of 96–97 %, but it incurred at least 8 additional hours of computation (total time 58 h) [12]. Furthermore, the original WGS_50_ methods featured genotyping of variants, rather than genotyping all genomic nucleotides. In particular, WGS_50_, did not distinguish reference genotypes from missing (uncalled) nucleotides.

**Table 2:** Comparison of the analytic performance of a conventional alignment and variant calling pipeline (GSNAP with GATK minus VQSR), with a novel, extremely rapid method (DRAGEN)

Sample	SBS_18_ yield (GB)	Site	Pipeline	Reads aligned	Alignments with mapping quality >20	Variants called	Mismatch rate	Indel rate	% Paired Reads	Strand balance	% Chimeric Reads	Rare, potentially pathogenic variants	Analytic sensitivity (GeT-RM or SNP array)	Analytic specificity (GeT-RM or SNP array)	Analytic sensitivity (full GIAB)	Analytic specificity (full GIAB)
NA12878	133	Essex	DRAGEN	99.4 %	95.48 %	4,782,970	0.0029	0.00017	99.55 %	0.500	0.69 %	658	99.93 %	99.87 %	99.69 %	99.99 %
			GSNAP/GATK-1.6/noVQSR	98.5 %	96.33 %	5,343,988	0.0056	0.00017	98.55 %	0.496	0.82 %	783	99.54 %	98.57 %	98.21 %	99.99 %
NA12878	65^a^	CMH	DRAGEN	97.7 %	91.31 %	4,633,357	0.0060	0.00023	99.18 %	0.501	1.89 %	775	99.42 %	99.46 %	98.63 %	99.99 %
			GSNAP/GATK-3.2/noVQSR	96.2 %	92.86 %	4,571,157	0.0079	0.00021	97.55 %	0.499	1.75 %	593	97.29 %	95.35 %	95.74 %	99.99 %
UDT_173	106	Essex	DRAGEN	99.5 %	94.92 %	4,742,150	0.0034	0.00020	99.80 %	0.500	1.12 %	620	96.13 %	97.74 %	n.a.	n.a.
			GSNAP/GATK-1.6/noVQSR	99.3 %	96.88 %	4,294,504	0.0034	0.00019	99.34 %	0.500	0.90 %	512	88.54 %	98.06 %	n.a.	n.a.

All runs were 18-h WGS. The NA12878 reference genotypes were NIST High Confidence calls from GeT-RM/NA12878.NIST-GIAB_v.2.18 (labeled ‘GeT-RM’) or the full GIAB dataset (labeled ‘full GIAB’). UDT_173 were results of hybridization to the Omni4 SNP array. GSNAP was version 2012.07.12, with default parameters, and GATK was version 1.6.13 or 3.2, without VQSR. DRAGEN was version 1.2. % paired, percentage of reads whose mate was also aligned; Strand balance, reads aligned to the forward strand divided by total reads aligned; % chimeric, percentage of chimeric alignments (mates >100 kb apart or on different chromosomes). ^a^Single flowcell

**Fig. 2:**
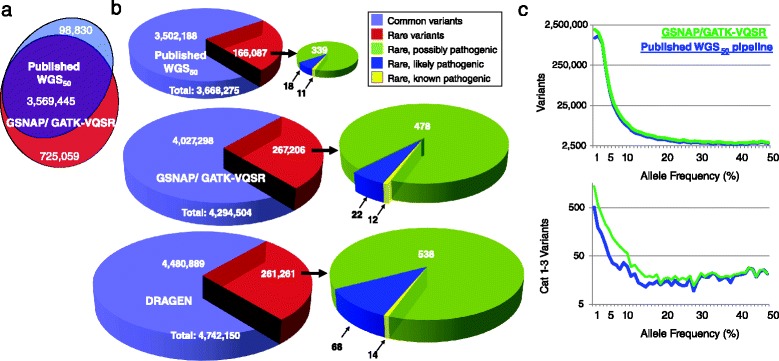
Improving the sensitivity of nucleotide variant identification for diagnosis of rare genetic diseases in approximately 35X human WGS. **a**. Venn diagram comparing nucleotide variants identified in WGS of sample UDT_173 (HiSeq 2500, 2 × 100 nt, 18-h run time) with previously disclosed methods for 50-h diagnostic WGS (Published WGS50 pipeline) [12], or with parameters described herein to improve sensitivity (GSNAP/GATK-VQSR). **b**. Pie charts showing the distribution of allele frequencies and pathogenicity of nucleotide variants reported by the three pipelines (Published WGS50, GSNAP/GATK-VQSR, and DRAGEN) in WGS of the same sample. Rare variants had allele frequencies <0.01, based on genomic sequences of approximately 3,000 internal samples. Previously reported disease causing variants are ACMG Category 1 mutations. Likely pathogenic variants are ACMG Category 2 variants (loss of initiation, premature stop codon, disruption of stop codon, whole gene deletion, frameshifting indel, disruption of splicing). Possibly pathogenic variants are ACMG Category 3 (non-synonymous substitution, in-frame indel, disruption of polypyrimidine tract, overlap with 5’ exonic, 5’ flank or 3’ exonic splice contexts, and intragenic mitochondrial variants). **c** Graphs of variant density versus variant allele frequency. Values for the two pipelines are plotted. Results represent the sum of approximately 40X WGS in three samples. Upper panel shows results for all variants. Lower panel shows results for ACMG Category 1–3 variants

The analytic sensitivity of rapid WGS increased to approximately 99.5 %, together with genotyping of all nucleotide positions, upon read alignment with GSNAP, and variant detection with GATK 1.6 or 3.2, with omission of variant quality score recalibration (VQSR) (Table 2, Fig. 2). The VQSR component of GATK reduced type 2 errors (β, false positives) in batched analyses of sequences in population research [28]. However, in singleton or trio diagnostic WGS, VQSR over-filtered novel, rare variants (allele frequency <1 %) that commonly cause monogenetic diseases (Fig. 2, Additional file 5: Figure S4).

**Fig. 3:**
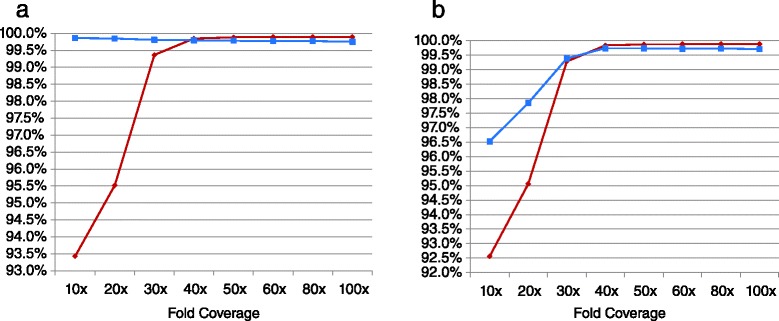
Variation in the sensitivity and specificity of nucleotide variant calls and genotypes as a product of the depth of the sequence. Several 2 × 100 nt runs of WGS of sample NA12878 were generated and the sensitivity (red diamonds) and specificity (blue squares) of variant calls (**a**) or genotypes (**b**) by GSNAP/GATK-VQSR were examined by comparison with a reference set (GeT-RM/NA12878.NIST-GIAB_v.2.18) at depth of coverage of 10X to 100X

Another rapid WGS variable we sought to optimize was the depth of coverage. It is a determinant of analytic sensitivity, analytic specificity, cost, and choice of sequencing instrument. The analytic sensitivity and specificity of the GSNAP/GATK-VQSR pipeline was calculated with 10-fold (32GB) to 100-fold (316 GB) coverage of sample NA12878 and compared with NIST reference genotypes (Fig. 3). Analytic sensitivity and specificity plateaued at approximately 40-fold (sensitivity 99.84 % and 99.85 %, and specificity 99.74 % and 99.85 %, for genotypes and variant calls, respectively). Further increases in depth of coverage were of no benefit for homozygous or heterozygous nucleotide variant calls.

### Optimizing the sensitivity and specificity of 26-h WGS

GSNAP/GATK-VQSR, while providing excellent sensitivity and specificity, was both costly and insufficiently rapid to be ideal for diagnosis in acutely ill neonates. Compute time on a 608 Intel Xeon core Linux cluster with 6 TB of DDR3 RAM and 20 TB SATA hard drives was 22.5 h. A number of alternative alignment and variant detection algorithms and hardware were evaluated. The most rapid and sensitive of these was DRAGEN v1.2 (Edico Genomics, La Jolla, CA, USA). Compute time on two 12-core Intel Xeon processors with hyper-threading technology (with 128 GB of RAM and 8 × 400 GB RAID-0 SSD on the staging disk) was 41 min for 40X WGS (Table 1). The analytic performance of DRAGEN and GSNAP/GATK-VQSR were compared in three SBS_18_ runs at two sites with varying sequence yield (Table 2). DRAGEN identified a similar number of genomic nucleotide variants to GSNAP/GATK-VQSR (averages of 4,719,492 and 4,736,550, respectively, in the three Caucasian genomes), and similar number of rare, potentially pathogenic variants (averages of 684 and 629 variants of ACMG categories 1–3 with allele frequencies <1 %, respectively, Table 2). However, DRAGEN provided both very rapid alignment and variant calling and slightly higher sensitivity and specificity than GSNAP/GATK 1.6 or 3.2 without VQSR (as high as 99.9 % for both; Tables 1 and 2).

**Fig. 4:**
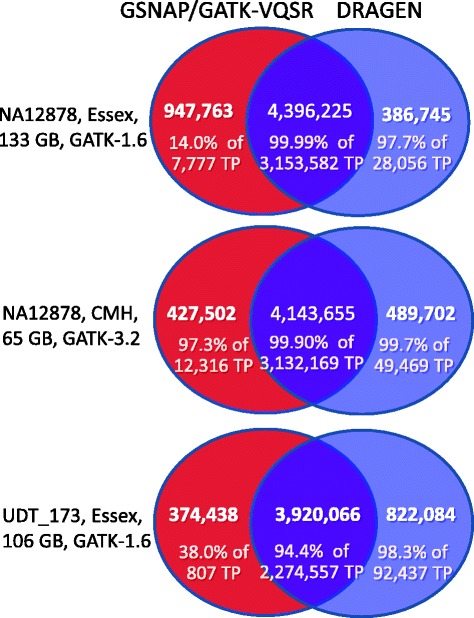
Comparison of the number and rate of true positive variant calls with GSNAP/GATK-VQSR and DRAGEN. The three samples and reference datasets are as in Tables 1 and 2. Numbers are variant calls. TP: Variants in the NA12878 CDC/GeT-RM clinical validation set in which true positive variant calls were made. %TP variants in the larger NIST/GIAB reference set were similar to those in the GeT-RM set (NA12878-essex, DRAGEN only 92.3 % of 143,385 TP, GATK only 19.8 % of 96,003 TP; NA12878-Gill, DRAGEN only 98.0 % of 1,335,504 TP, GATK only 91.8 % of 58,571 TP)

Recently, it has been recognized that various alignment and variant calling pipelines identify overlapping but distinct sets of true-positive nucleotide positives [12, 29]. Therefore, the overlap of variants identified by GSNAP/GATK-VQSR and DRAGEN was examined in the three genome sequences (Fig. 4). GSNAP/GATK-VQSR identified 89.3 % of the combined total 15,908,180 variants detected by the two pipelines, whereas DRAGEN identified 89.0 %. Among the 10.7 % variants uniquely identified by GSNAP/GATK-VQSR, 40.9 % of variants that could be assessed through comparison to a truth set were true positives, whereas 98.6 % of the 10.7 % uniquely identified by DRAGEN were true positives. These findings were reproduced both in the smaller CDC/GeT-RM clinical validation dataset from reference sample NA12878 and in the full GIAB dataset (Table 2, Fig. 4). GATK version 3.2 outperformed version 1.6 in this comparison (97.3 % true positives with version 3.2, versus an average of 27.3 % with version 1.6, Fig. 4). Thus, maximum analytic sensitivity was accomplished by combining variant calls of the DRAGEN and GSNAP/GATK 3.2-VQSR pipelines. However, where variant genotypes differed between the two pipelines, the resolution would likely require visual inspection of read alignments in diagnostic candidate genes.

These data support the use of multiple alignment and calling algorithms for maximal sensitivity, and highlight deficiencies in the NA12878 reference datasets.

## Discussion

Here we have described methods for rapid, medical WGS (version 2 STATseq), with greater analytic sensitivity (99.5 % in a 40X genome), faster time to result, and improved scalability. Twenty-six hours was the shortest elapsed time from receipt of a blood sample to diagnosis of a genetic disease. Twenty-six hours was possible when readily apparent upon application of a standardized set of variant filters using VIKING software and integration of an automated differential diagnosis based on SSAGA or Phenomizer software. It assumed no time interval between steps in the protocol. Maximum analytic sensitivity was achieved by combining variant calls of the DRAGEN and GSNAP/GATK 3.2-VQSR pipelines. The most significant innovations were as follows: First, approximately 18–21 h to generate 30–47-fold, 2 × 100 nt SBS with a modified Illumina HiSeq 2500. Second, approximately 1 h for read alignment, variant calling, annotation, and interpretation. Importantly, the methods were replicated both in a research laboratory (Illumina, Essex) and in a genome center in a children’s hospital (CM-KC).

In addition to speed, the methods described herein enable scaling of medical WGS to approximately 350 samples per year per sequencing instrument. The DRAGEN alignment and variant calling hardware and software has specifications which are likely to make genome sequencing practicable in many hospital laboratories, such as reducing the need for cloud computing or a large local cluster. The VIKING software greatly alleviates the burden of genome analysis and interpretation and allows common inheritance modes to be rapidly examined. When a diplotype of likely pathogenic variants is observed in a gene that ranks high on the differential diagnosis, interpretation can be performed in minutes. Ruling out a genetic diagnosis or making a diagnosis in situations of novel phenotype expansion, however, is an arduous process, involving hours of effort by a highly experienced, laboratory geneticist, even when assisted by software.

### Optimizing sensitivity

Diagnostic sensitivity is the single most important attribute for medical WGS. Here we examined a surrogate, namely analytic sensitivity for nucleotide variants; 99.9 % analytic sensitivity and specificity of genome-wide genotypes was obtained with high quality, 47X genome sequence and the DRAGEN pipeline in a 26-h format. Notably, this figure reflects both substitutions and indels (of size up to 469 nt). Possibly more remarkable was 99.4 % analytic sensitivity and specificity with 20X genome sequence and the same methods. Furthermore, analytic sensitivity was further increased when two alignment algorithms and variant callers are used, as has been suggested [12, 29]. Herein, we achieved maximum analytic sensitivity by combining variant calls of the DRAGEN and GSNAP/GATK 3.2-VQSR pipelines. In a diagnostic use-case, the issue of which two conflicting genotypes to retain at sites where variant genotypes differ between the two pipelines may be solved simply by retention of the more pathogenic genotype. Greater sensitivity resulted in a remarkable increase in rare variants, which were being over-filtered by conventional pipelines. Thus, the approximately 2.8 billion nucleotides of genomes that can be genotyped with paired, short reads contain approximately 5 million nucleotide variants in individuals of northern European ancestry, and approximately 6 million in those of African ancestry. The ultra-sensitive GSNAP/GATK-VQSR pipeline has been in routine use in a clinical laboratory with Sanger confirmation of hundreds of diagnostic genotypes [6, 11] (Saunders et al., unpublished). This experience has confirmed the results reported herein – namely that analytic specificity remains adequate for clinical use despite such sensitivity. In short, we believe that we, as a community, been missing many variants due to the limitations of our software algorithms. However, in contrast to analytic sensitivity, further work is needed to determine whether a two-pipeline method improves diagnostic yield sufficiently to be cost effective in light of decreased specificity.

For greatest usefulness as a clinical diagnostic tool, WGS must genotype all genomic sites, whether reference or variant calls. In this manner, WGS can be used both for diagnosis and to rule out treatable genetic diagnoses. From a clinician perspective, ruling out treatable genetic disease diagnoses or diseases with benign prognosis is paramount for clinical decision-making. In particular, in acutely ill infants in a NICU setting, end-of-life decisions are common, with most deaths resulting from withholding or withdrawing care after careful weighting of the prognosis by the care team and family [11, 30]. Notably, the methods described here assign values to approximately 2.8 billion nucleotides, whether variant genotypes, reference genotypes, or no call. With further software development, it should be possible to generate an automatic report of the completeness of genotyping of all protein coding nucleotides and intron-exon boundaries of relevant disease genes with defined coverage and quality scores, and, thereby, in the future, to ‘rule out’ specific diagnoses.

### Limitations of rapid WGS

While medical WGS is becoming increasingly robust, especially relative to exome sequencing, it is appropriate to highlight its current analytic limitations for genetic disease diagnosis. The analytic sensitivity for variants other than nucleotide variants is too low for use as a stand-alone clinical test. Notable deficiencies of paired, short-read WGS are analytic sensitivity and specificity for pathogenic structural variations and triplet repeat expansions. Phenotype-associated genes with highly homologous pseudogenes require custom software solutions to disambiguate variants mapping to the gene or pseudogene. The biggest limitation for medical WGS and exome sequencing, however, is the interpretation of variants of uncertain significance (VUS). For these reasons, genetic disease diagnosis will continue to require multiple types of testing, including functional and confirmatory testing, for the foreseeable future.

Another current limitation of WGS_26_ is that it is a research method, and confirmatory testing of causative genotypes, which is typically required for diagnostic reporting, takes at least two days. Upon protocol validation to meet CLIA and CAP guidelines for laboratory developed tests (LDTs), however, the requirement for confirmatory testing will be decided on a case-by-case basis by an accredited laboratory director. Over the next several years, however, some type of FDA approval will also be required for high complexity LDTs, such as medical WGS. A pre-investigational device exemption inquiry was made for clinical research use of WGS_50_ for diagnosis of genetic diseases in acutely ill infants in our level IV (regional) NICU. Encouragingly, the FDA conferred non-significant risk status for these methods for research use in this setting.

A third limitation of current WGS is lack of comprehensive negative predictive value. On a gene-by-gene basis, current WGS allows visual inspection for gaps in exonic or intronic coverage. Thus, where a single diagnosis – such as MSUD – must be ruled out, this can readily be accomplished. A significant advantage of WGS over exome sequencing is more complete coverage. In particular, exome sequencing tends to suffer loss of coverage for first exons. In addition to imperfect analytic sensitivity, however, diagnostic sensitivity is limited by lack of knowledge of all pathogenic variants. In particular, pathogenic intronic and regulatory variants are under-represented in clinical databases, and, in contrast to exonic variants of uncertain significance, cannot not yet robustly assayed by *in silico* pathogenicity prediction tools.

It is interesting to speculate what the fastest time to diagnostic result might be with current WGS technology. Technically, a substantial reduction in sample preparation time from 7.5 h should be possible. With customized robotics, these pre-analytic steps should be feasible in 2 h. SBS should be possible in approximately 10 h with 2 × 50 cycles. *In silico* modeling suggests that analytic sensitivity and specificity for nucleotide variants would remain >95 % with such read lengths. Stranneheim et al. have described pulsed whole genome sequencing with analysis of results iteratively at 35, 50, 75, and 100 cycles [31]. When combined with the DRAGEN system, there is the possibility of near real-time analysis of results whereby sequencing continues until a diagnosis is achieved. While further reductions in time to result may seem pedantic, sub-24 h time to result can be material since medical rounds typically occur once a day. Thus, return of results between 07:00–11:00 allows their significance to be discussed by the whole medical team when fresh. Off-hours results are returned to an on-duty physician who is likely to need specialist consultation.

An unsolved need for medical WGS_26_ is sample multiplexing, both to lower the cost of testing and to allow trios to be analyzed simultaneously. Sequencing of parent–infant trios is necessary for genetic disease diagnosis since the most common mechanism of causative mutations is *de novo*. WGS_26_ is performed one sample at a time (dual flowcells) at a reagent cost of $6,500, which is more than eight-fold greater than WGS on a HiSeq X. WGS_26_ sequencer depreciation is approximately $714 per genome at full capacity (350 genomes per year), compared with $137 on a HiSeq X. Technician cost is similar (approximately $70 per genome). The cost of computation and automated analysis varies widely with scale, but around a median of approximately $100 per genome. Interpretation and reporting is in the range of $70–$700 depending on the number and types of variants identified in a trio. Thus, cost is a significant barrier to broad adoption of WGS_26_, particularly given the mark-ups in price that are commonly employed in the US medical system to offset negotiated discounts or lack of payment. An attractive compromise between cost and speed is the HiSeq X configured to perform approximately 450 GB of 2 × 75 nt sequencing in a trio in 33 h on a single flowcell, for a total turnaround time of approximately 41 h. An alternative is rapid exome sequencing (WES), using the WGS_26_ software and hardware. With ongoing improvements in the hybridization kinetics of exome capture probes, and in the representation of all exons, a 36-h, 100X WES of three trios per $6,500 run should be feasible.

Finally, it is worth briefly mentioning the medical applications that currently may benefit from a 26-h, rather than a less costly 6-day, medical genome. These are applications that have a relatively high likelihood of guiding acute medical decisions in clinical situations where a delay is likely to result in significant morbidity or mortality. Currently, the best defined such application is in the differential diagnosis of certain single gene diseases. One example is maple syrup urine disease (MSUD, OMIM #248600), in which irritability and poor feeding typically occur within 48 h of delivery. Lethargy, intermittent apnea, opisthotonus, and stereotyped movements are evident by day of life 4. Diagnosis and institution of treatment before the onset of these neurologic signs significantly reduces the lifetime risk of mental illness and global functional impairment [32, 33]. Mass spectrometry of blood at 48 h of life is used to screen infants for MSUD in newborn screening programs. While typically positive in affected newborns, results may be delayed until after onset of neurologic signs or an initial screen may be falsely negative if the newborn has not fed appropriately after delivery.

Our initial clinical experience with rapid WGS involved 35 parent–infant trios [6, 11, 12]. All infants were acutely ill, aged less than 4 months at the time of enrollment, had a suspected genetic cause of disease, and lacked a molecular diagnosis. Clinical features in these infants were typically apparent at birth. Rapid WGS provided a genetic diagnosis in 20 (57 %) infants. In nine (45 %) infants receiving a diagnosis, the condition had not been considered in the differential diagnosis at the time of enrollment. Thirteen (65 %) diagnoses were noted to have acute clinical utility, and four (20 %) diagnoses had strongly favorable effects on management. However, six (30 %) diagnosed infants were started on palliative care and 120-day mortality was 57 %. A randomized, prospective clinical study of rapid WGS is now in progress to ascertain the extensibility of these results to broad NICU populations. Clearly, while the application of rapid WGS for NICU diagnosis of genetic disease appears tremendously promising, translating diagnoses into effective precision medicine is in its infancy.

## Conclusions

Twenty-six-hour STATseq appears to be an appropriate strategy for acutely ill patients with potentially actionable genetic diseases. Having demonstrated improved analytic performance of version 2 STATseq, and time to result of 26 h, the next step is to retrospectively analyze the diagnostic yield of these methods, particularly in cases where no diagnostic diplotype was identified by conventional WGS.

### Data and materials

The genomic sequence data for this study have been deposited in the database dbGAP with accession number phs000564. The CM-KC software described herein is in development for availability as freeware for research use.

## Additional files

Additional file 1: Figure S1. Screen-shots demonstrating the functionality of SSAGA. A. The clinical feature entry page. Synonyms for each feature are entered in the top left box. Upon entry, a list of matching HPO terms is displayed. The appropriate HPO term is selected and added to the patient’s feature list in the box on the right. This is performed for each clinical feature. In this case, patient CMH672ref, the patient had 11 clinical features that included neonatal seizures and a characteristic facies. B. Upon clicking the ‘Get Diagnosis’ button, the list of all matching diseases is generated. In this case, the differential diagnosis had 1,136 rows, representing 597 genes, of which 222 matched two or more clinical features. (PDF 240 kb) Additional file 2: Figure S2. A screen-shot of the warehouse annotation and curation data for a genomic variant. Right clicking a variant row in VIKING opens a menu that includes a link-out to the CMH Variant Warehouse, which contains automated annotation data from RUNES (ACMG-type variant category, CM-KC allele frequency, homozygous and heterozygous status in other samples, BLOSUM score, SIFT score, and PolyPhen2 score), Entrez Gene, HGMD, ClinVar, COSMIC, and manual curation data for that variant (if available). Highlighted values represent hyperlinks to additional information. (PDF 210 kb) Additional file 3: Figure S3. A screen-shot of VIKING showing variants identified by DRAGEN in WGS26 of sample UDT_103, a patient with a molecular diagnosis of familial hemophagocytic lymphohistiocytosis type 3. Variants are displayed as rows in the right hand panel. The variant attributes are displayed by columns in the right hand panel. The bottom left panel permits selection of the variant attributes to be displayed. The following filters have been applied to the UDT_103 variant set (top left panel): Filtering to retain variants of ACMG-type categories 1–3, with an allele frequency in the CM-KC database of <0.1 %, that fit a recessive inheritance pattern (hemizygous, homozygous or compound heterozygous), and limited to OMIM genes. Twenty-six variants meet these filters. Ten of these have been manually interpreted and flagged as as ‘likely benign’ by the operator, by highlighting in light blue. Two variants have been interpreted as ‘likely pathogenic’ by the operator, by highlighting in light brown. The column abbreviations are: Chr, Chromosome. Start, variant start genomic nucleotide. HGVS_c, HGVS nomenclature for nucleotide variants in transcripts. HGVS_p, HGVS nomenclature for corresponding amino acid changes in peptides. ACMG, ACMG-type pathogenicity category. MAF, CM-KC allele frequency, rsID, dbSNP accession number. dbSNP, dbSNP allele frequency. Zyg…, Zygosity. Quality, read alignment quality. Read, number of reads calling variant/number of reads aligned at that nucleotide. (PDF 164 kb) Additional file 4: Table S1. Comparison of the metrics of sequence yield and quality of 18-h and 26-h WGS (HiSeq 2500 2 × 100 nt rapid-run mode). a, R2 refers to read 2. Eighteen-hour runs had marginally better quality than 26-h runs, given slight differences in average cluster density. This might have been due to the shorter time of slide exposure to laser light and lesser loss in reagent stability. b. Comparison of 18-h and 26-h WGS metrics (HiSeq 2500 2 × 100 nt rapid-run mode), showing correlations between cluster density and metrics of sequence yield and quality. Cluster density explained much of the variability in yield, quality score, error rate, and % reads passing filter. (PDF 23 kb) Additional file 5: Figure S4. Examination of the sensitivity and accuracy of nucleotide variant genotype calls in WGS with the published WGS50 and GSNAP/GATK-VQSR pipelines. A. Comparison of the sensitivity and accuracy of all nucleotide variant calls. B. Comparison of the accuracy of variants that were uniquely called by the GSNAP/GATK-VQSR. WGS was performed using the HiSeq 2500 with 2 × 100 cycles and 18-h run time. The sample UDT_173 genotype “truth set” was from hybridization to the Omni4 SNP array. The NA12878 ‘truth set’ was from ftp://ftp-trace.ncbi.nih.gov/giab/ftp/data/NA12878/variant_calls/NIST26. (PDF 213 kb)
