# Supplementary material for: A 26-hour system of highly sensitive whole genome sequencing for emergency management of genetic diseases
Source: Genome Med. 2015 Sep 30;7:100. doi: 10.1186/s13073-015-0221-8 (PMC4588251; doi:10.1186/s13073-015-0221-8)
Supplement: Additional file 1: Figure S1. — Screen-shots demonstrating the functionality of SSAGA. A. The clinical feature entry page. Synonyms for each feature are entered in the top left box. Upon entry, a list of matching HPO terms is displayed. The appropriate HPO term is selected and added to the patient’s feature list in the box on the right. This is performed for each clinical feature. In this case, patient CMH672ref, the patient had 11 clinical features that included neonatal seizures and a characteristic facies. B. Upon clicking the ‘Get Diagnosis’ button, the list of all matching diseases is generated. In this case, the differential diagnosis had 1,136 rows, representing 597 genes, of which 222 matched two or more clinical features. (PDF 240 kb) [file 13073_2015_221_MOESM1_ESM.pdf]

a

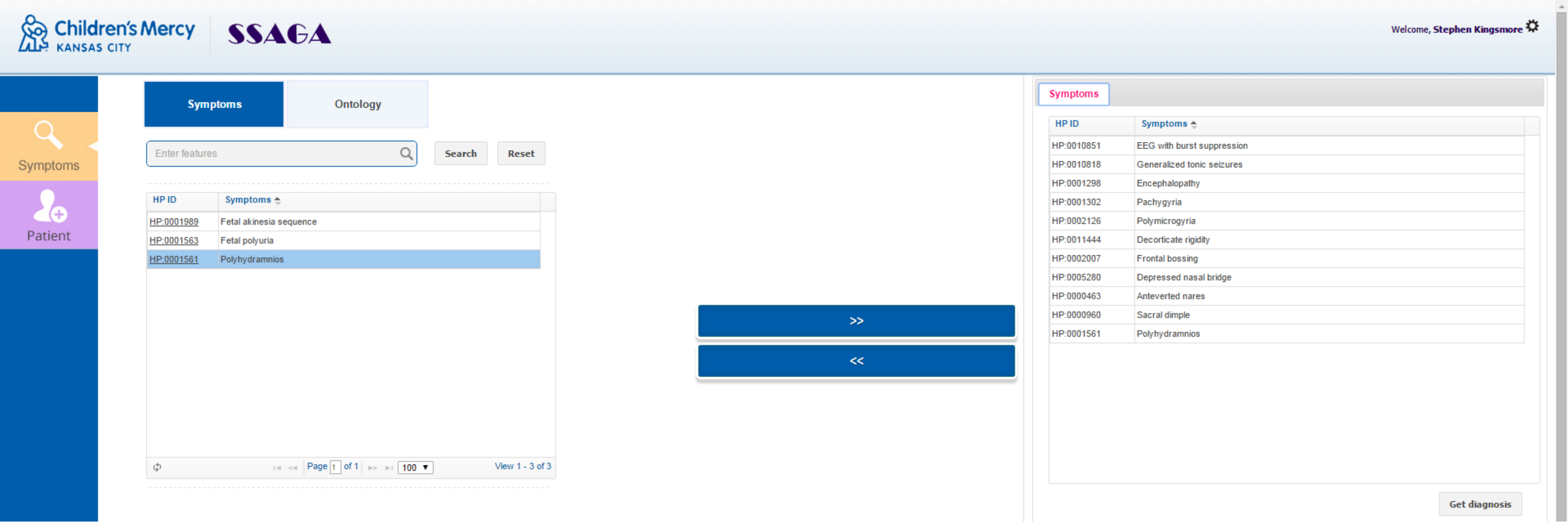
Children's Mercy
KANSAS CITY
SSAGA
Welcome, Stephen Kingsmore
Symptoms
Ontology
Enter features
Search
Reset
Symptoms
Patient
HP ID
Symptoms
HP:0001989 Fetal akinesia sequence
HP:0001563 Fetal polyuria
HP:0001561 Polyhydramnios
Page 1 of 1
100
View 1 - 3 of 3
>>
<<
Symptoms
HP ID
Symptoms
HP:0010851 EEG with burst suppression
HP:0010818 Generalized tonic seizures
HP:0001298 Encephalopathy
HP:0001302 Pachygyria
HP:0002126 Polymicrogyria
HP:0011444 Decorticate rigidity
HP:0002007 Frontal bossing
HP:0005280 Depressed nasal bridge
HP:0000463 Anteverted nares
HP:0000960 Sacral dimple
HP:0001561 Polyhydramnios
Get diagnosis

b

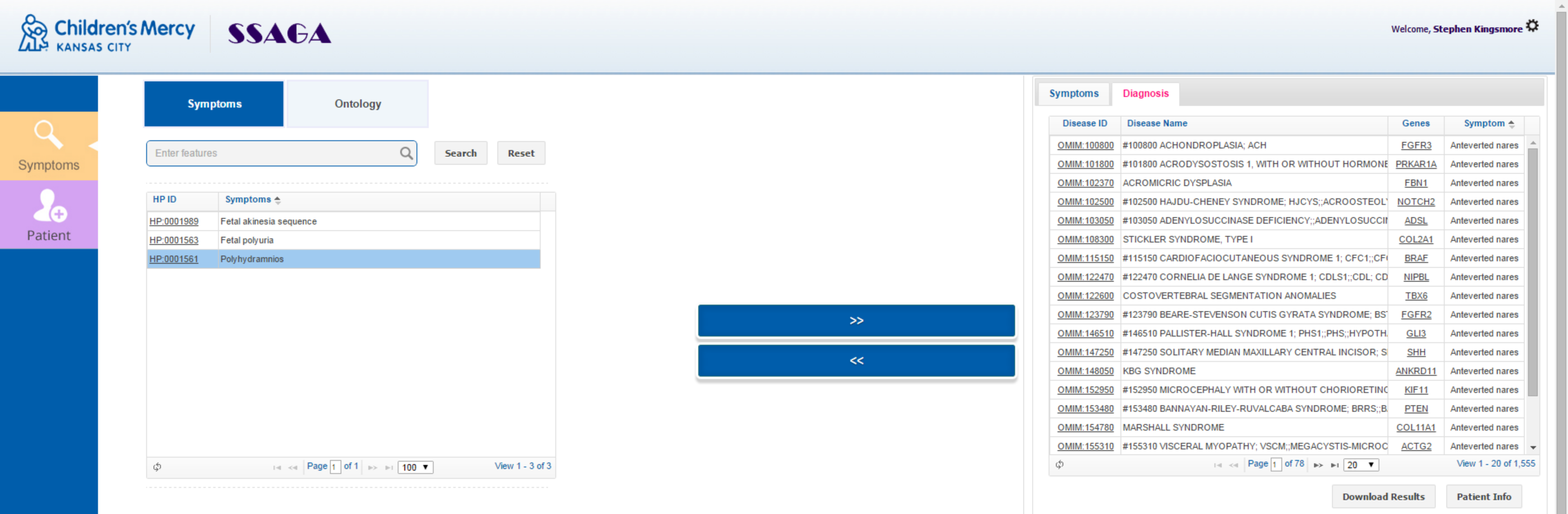
Children's Mercy
KANSAS CITY
SSAGA
Welcome, Stephen Kingsmore
Symptoms
Ontology
Enter features
Search
Reset
Symptoms
Patient
HP ID
Symptoms
HP:0001989 Fetal akinesia sequence
HP:0001563 Fetal polyuria
HP:0001561 Polyhydramnios
Page 1 of 1
100
View 1 - 3 of 3
>>
<<
Symptoms
Diagnosis
Disease ID
Disease Name
Genes
Symptom
OMIM:100800 #100800 ACHONDROPLASIA; ACH FGFR3 Anteverted nares
OMIM:101800 #101800 ACRODYSOSTOSIS 1, WITH OR WITHOUT HORMONE PRKAR1A Anteverted nares
OMIM:102370 ACROMICRIC DYSPLASIA FBN1 Anteverted nares
OMIM:102500 #102500 HAJDU-CHENEY SYNDROME; HJCYS;;ACROOSTEOL NOTCH2 Anteverted nares
OMIM:103050 #103050 ADENYLOSUCCINASE DEFICIENCY;;ADENYLOSUCCI ADSL Anteverted nares
OMIM:108300 STICKLER SYNDROME, TYPE I COL2A1 Anteverted nares
OMIM:115150 #115150 CARDIOFACIOCUTANEOUS SYNDROME 1; CFC1;;CF BRAF Anteverted nares
OMIM:122470 #122470 CORNELIA DE LANGE SYNDROME 1; CDLS1;;CDL; CD NIPBL Anteverted nares
OMIM:122600 COSTOVERTEBRAL SEGMENTATION ANOMALIES TBX6 Anteverted nares
OMIM:123790 #123790 BEARE-STEVENSON CUTIS GYRATA SYNDROME; BS FGFR2 Anteverted nares
OMIM:146510 #146510 PALLISTER-HALL SYNDROME 1; PHS1;;PHS;;HYPOTH GLI3 Anteverted nares
OMIM:147250 #147250 SOLITARY MEDIAN MAXILLARY CENTRAL INCISOR; S SHH Anteverted nares
OMIM:148050 KBG SYNDROME ANKRD11 Anteverted nares
OMIM:152950 #152950 MICROCEPHALY WITH OR WITHOUT CHORIORETINO KIF11 Anteverted nares
OMIM:153480 #153480 BANNAYAN-RILEY-RUVALCABA SYNDROME; BRRS;;B PTEN Anteverted nares
OMIM:154780 MARSHALL SYNDROME COL11A1 Anteverted nares
OMIM:155310 #155310 VISCERAL MYOPATHY; VSCM;;MEGACYSTIS-MICROC ACTG2 Anteverted nares
Page 1 of 78
20
View 1 - 20 of 1,555
Download Results
Patient Info
