# Supplementary material for: A 26-hour system of highly sensitive whole genome sequencing for emergency management of genetic diseases
Source: Genome Med. 2015 Sep 30;7:100. doi: 10.1186/s13073-015-0221-8 (PMC4588251; doi:10.1186/s13073-015-0221-8)
Supplement: Additional file 2: Figure S2. — A screen-shot of the warehouse annotation and curation data for a genomic variant. Right clicking a variant row in VIKING opens a menu that includes a link-out to the CMH Variant Warehouse, which contains automated annotation data from RUNES (ACMG-type variant category, CM-KC allele frequency, homozygous and heterozygous status in other samples, BLOSUM score, SIFT score, and PolyPhen2 score), Entrez Gene, HGMD, ClinVar, COSMIC, and manual curation data for that variant (if available). Highlighted values represent hyperlinks to additional information. (PDF 210 kb) [file 13073_2015_221_MOESM2_ESM.pdf]

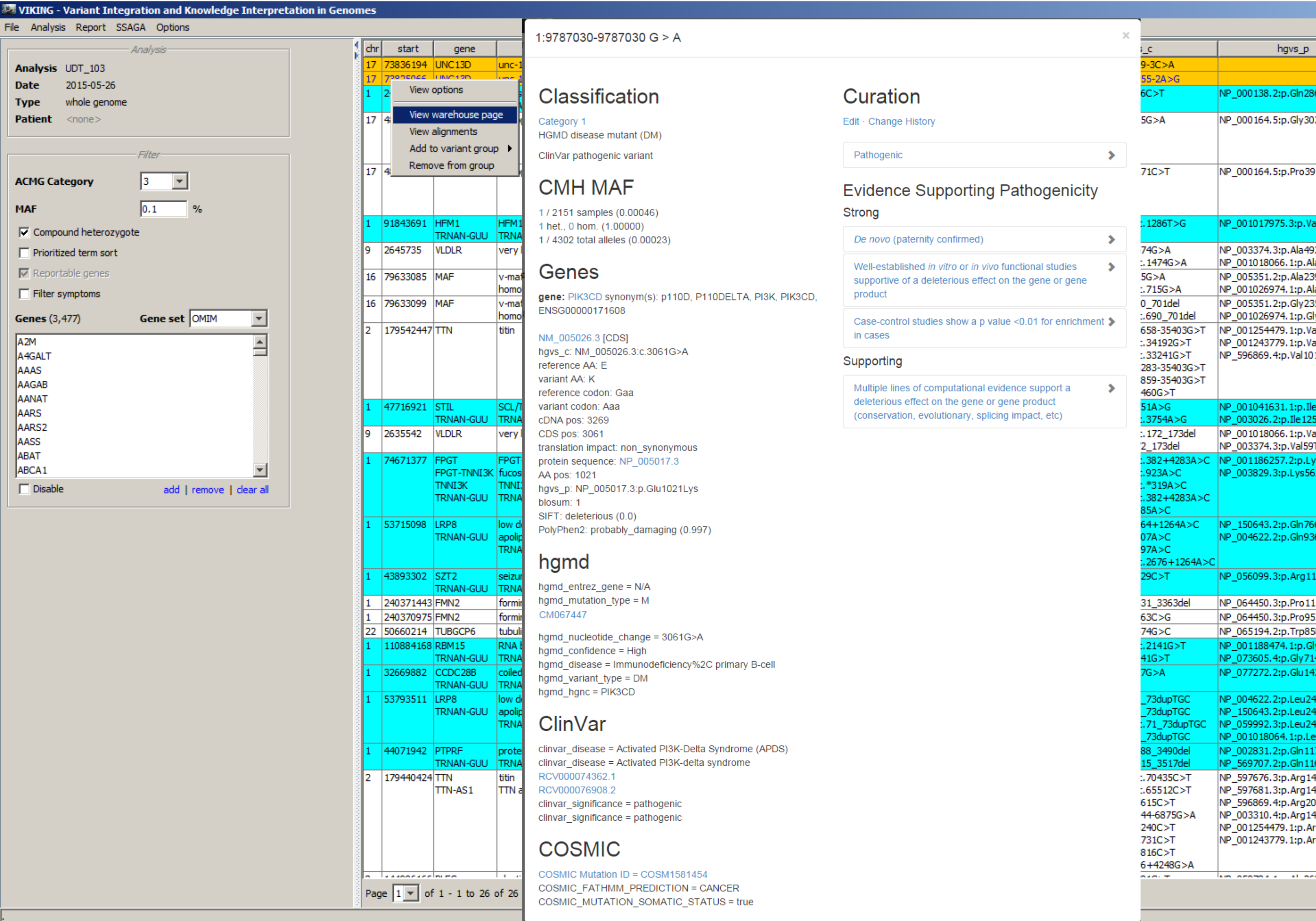
VIKING - Variant Integration and Knowledge Interpretation in Genomes
File
Analysis
Report
SSAGA
Options
Analysis
Analysis UDT_103
Date 2015-05-26
Type whole genome
Patient <none>
Filter
ACMG Category 3
MAF 0.1 %
Compound heterozygote
Prioritized term sort
Reportable genes
Filter symptoms
Genes (3,477)
Gene set OMIM
A2M
A4GALT
AAAS
AAGAB
AANAT
AARS
AARS2
AASS
ABAT
ABCA1
Disable
add | remove | clear all
chr
start
gene
hgvs_p
View options
View warehouse page
View alignments
Add to variant group
Remove from group
Page 1 of 1 - 1 to 26 of 26
1:9787030-9787030 G > A
Classification
Category 1
HGMD disease mutant (DM)
ClinVar pathogenic variant
CMH MAF
1 / 2151 samples (0.00046)
1 het., 0 hom. (1.00000)
1 / 4302 total alleles (0.00023)
Genes
gene: PIK3CD synonym(s): p110D, P110DELTA, PI3K, PIK3CD, ENSG00000171608
NM_005026.3 [CDS]
hgvs_c: NM_005026.3:c.3061G>A
reference AA: E
variant AA: K
reference codon: Gaa
variant codon: Aaa
cDNA pos: 3269
CDS pos: 3061
translation impact: non_synonymous
protein sequence: NP_005017.3
AA pos: 1021
hgvs_p: NP_005017.3:p.Glu1021Lys
blosum: 1
SIFT: deleterious (0.0)
PolyPhen2: probably_damaging (0.997)
hgmd
hgmd_entrez_gene = N/A
hgmd_mutation_type = M
CM067447
hgmd_nucleotide_change = 3061G>A
hgmd_confidence = High
hgmd_disease = Immunodeficiency%2C primary B-cell
hgmd_variant_type = DM
hgmd_hgnc = PIK3CD
ClinVar
clinvar_disease = Activated PI3K-Delta Syndrome (APDS)
clinvar_disease = Activated PI3K-delta syndrome
RCV000074362.1
RCV000076908.2
clinvar_significance = pathogenic
clinvar_significance = pathogenic
COSMIC
COSMIC Mutation ID = COSM1581454
COSMIC_FATHMM_PREDICTION = CANCER
COSMIC_MUTATION_SOMATIC_STATUS = true
Curation
Edit · Change History
Pathogenic
Evidence Supporting Pathogenicity
Strong
De novo (paternity confirmed)
Well-established in vitro or in vivo functional studies supportive of a deleterious effect on the gene or gene product
Case-control studies show a p value <0.01 for enrichment in cases
Supporting
Multiple lines of computational evidence support a deleterious effect on the gene or gene product (conservation, evolutionary, splicing impact, etc)
