# Supplementary material for: A 26-hour system of highly sensitive whole genome sequencing for emergency management of genetic diseases
Source: Genome Med. 2015 Sep 30;7:100. doi: 10.1186/s13073-015-0221-8 (PMC4588251; doi:10.1186/s13073-015-0221-8)
Supplement: Additional file 3: Figure S3. — A screen-shot of VIKING showing variants identified by DRAGEN in WGS26 of sample UDT_103, a patient with a molecular diagnosis of familial hemophagocytic lymphohistiocytosis type 3. Variants are displayed as rows in the right hand panel. The variant attributes are displayed by columns in the right hand panel. The bottom left panel permits selection of the variant attributes to be displayed. The following filters have been applied to the UDT_103 variant set (top left panel): Filtering to retain variants of ACMG-type categories 1–3, with an allele frequency in the CM-KC database of <0.1 %, that fit a recessive inheritance pattern (hemizygous, homozygous or compound heterozygous), and limited to OMIM genes. Twenty-six variants meet these filters. Ten of these have been manually interpreted and flagged as as ‘likely benign’ by the operator, by highlighting in light blue. Two variants have been interpreted as ‘likely pathogenic’ by the operator, by highlighting in light brown. The column abbreviations are: Chr, Chromosome. Start, variant start genomic nucleotide. HGVS_c, HGVS nomenclature for nucleotide variants in transcripts. HGVS_p, HGVS nomenclature for corresponding amino acid changes in peptides. ACMG, ACMG-type pathogenicity category. MAF, CM-KC allele frequency, rsID, dbSNP accession number. dbSNP, dbSNP allele frequency. Zyg…, Zygosity. Quality, read alignment quality. Read, number of reads calling variant/number of reads aligned at that nucleotide. (PDF 164 kb) [file 13073_2015_221_MOESM3_ESM.pdf]

VIKING - Variant Integration and Knowledge Interpretation in Genomes

File Analysis Report SSAGA Options

*Analysis*

**Analysis** UDT_103
**Date** 2015-05-26
**Type** whole genome
**Patient** <none>

*Filter*

**ACMG Category** 3
**MAF** 0.1 %
☑ Compound heterozygote
☐ Prioritized term sort
☑ Reportable genes
☐ Filter symptoms

**Genes** (3,477) **Gene set** OMIM

A2M
A4GALT
AAAS
AAGAB
AANAT
AARS
AARS2
AASS
ABAT
ABCA1

☐ Disable add | remove | clear all

**View Options**

☑ HGVS_c ☑ HGVS_p ☐ AA change
☑ gene ☑ gene full name ☑ OMIM phenotype
☑ chr ☑ start ☐ stop
☑ ACMG category ☐ ACMG note ☐ inheritance
☑ rsID ☑ dbSNP MAF ☑ RUNES MAF
☐ translation impact ☐ splicing impact ☐ transcript
☑ zygosity ☐ variant allele ☐ reference allele
☐ term count ☐ terms ☐ gene report category
☐ het. pct ☐ sample pct ☐ has curation
☑ quality ☑ reads

Apply Cancel

| chr | start | gene | geneFullName | omimPhenotype | hgvs_c | hgvs_p | acmg | maf | rsId | dbSnp... | zyg... | quality | reads |
|---|---|---|---|---|---|---|---|---|---|---|---|---|---|
| 17 | 73836194 | UNC13D | unc-13 homolog D (C. elegans) | Hemophagocytic lymphohistiocytosis, familial, 3 | NM_199242.2:c.859-3C>A | | 3 | 0.0001 | | 0.0 | het. | 99.0 | 18 / 35 |
| 17 | 73825066 | UNC13D | unc-13 homolog D (C. elegans) | Hemophagocytic lymphohistiocytosis, familial, 3 | NM_199242.2:c.2955-2A>G | | 2 | 0.0001 | | 0.0 | het. | 99.0 | 16 / 31 |
| 1 | 24180963 | FUCA1<br>TRNAN-GUU | fucosidase, alpha-L- 1, tissue<br>TRNAN-GUU | Fucosidosis | NM_000147.4:c.856C>T | NP_000138.2:p.Gln286Ter | 2 | 0.0001 | | 0.0 | het. | 99.0 | 24 / 41 |
| 17 | 4836804 | GP1BA | glycoprotein Ib (platelet), alpha polypeptide | Bernard-Soulier syndrome, type A1 (recessive); {Nonarteritic anterior ischemic optic neuropathy, susceptibility to}; Bernard-Soulier syndrome, type A2 (dominant); von Willebrand disease, platelet-type | NM_000173.5:c.905G>A | NP_000164.5:p.Gly302Asp | 3 | 0.0002 | rs185903059 | 9.183E-4 | het. | 99.0 | 27 / 50 |
| 17 | 4837070 | GP1BA | glycoprotein Ib (platelet), alpha polypeptide | Bernard-Soulier syndrome, type A1 (recessive); {Nonarteritic anterior ischemic optic neuropathy, susceptibility to}; Bernard-Soulier syndrome, type A2 (dominant); von Willebrand disease, platelet-type | NM_000173.5:c.1171C>T | NP_000164.5:p.Pro391Ser | 3 | 0.0009 | rs147455264 | 0.005969 | het. | 99.0 | 17 / 30 |
| 1 | 91843691 | HFM1<br>TRNAN-GUU | HFM1, ATP-dependent DNA helicase homolog (S. cerevisiae)<br>TRNAN-GUU | Premature ovarian failure 9 | NM_001017975.3:c.1286T>G | NP_001017975.3:p.Val429Gly | 3 | 0.0002 | | 0.0 | het. | 99.0 | 18 / 47 |
| 9 | 2645735 | VLDLR | very low density lipoprotein receptor | Cerebellar hypoplasia and mental retardation with or without quadrupedal locomotion 1 | NM_003383.3:c.1474G>A<br>NM_001018056.1:c.1474G>A | NP_003374.3:p.Ala492Thr<br>NP_001018066.1:p.Ala492Thr | 3 | 0.0001 | rs141757226 | 0.0 | het. | 99.0 | 27 / 52 |
| 16 | 79633085 | MAF | v-maf avian musculoaponeurotic fibrosarcoma oncogene homolog | Cataract 21, multiple types | NM_005360.4:c.715G>A<br>NM_001031804.2:c.715G>A | NP_005351.2:p.Ala239Thr<br>NP_001026974.1:p.Ala239Thr | 3 | 0.0002 | | 0.0 | het. | 13.0 | 5 / 6 |
| 16 | 79633099 | MAF | v-maf avian musculoaponeurotic fibrosarcoma oncogene homolog | Cataract 21, multiple types | NM_005360.4:c.690_701del<br>NM_001031804.2:c.690_701del | NP_005351.2:p.Gly235_Gly238del<br>NP_001026974.1:p.Gly235_Gly238del | 3 | 0.0000 | | 0.0 | het. | 74.0 | 2 / 4 |
| 2 | 179542447 | TTN | titin | Cardiomyopathy, familial hypertrophic, 9; Cardiomyopathy, dilated, 1G; Tibial muscular dystrophy, tardive; Muscular dystrophy, limb-girdle, type 2J; Myopathy, proximal, with early respiratory muscle involvement; Myopathy, early-onset, with fatal cardiomyopathy | NM_133432.3:c.13658-35403G>T<br>NM_001267550.1:c.34192G>T<br>NM_001256850.1:c.33241G>T<br>NM_003319.4:c.13283-35403G>T<br>NM_133437.3:c.13859-35403G>T<br>NM_133378.4:c.30460G>T | NP_001254479.1:p.Val11398Phe<br>NP_001243779.1:p.Val11081Phe<br>NP_596869.4:p.Val10154Phe | 3 | 0.0002 | | 0.0 | het. | 99.0 | 27 / 62 |
| 1 | 47716921 | STIL<br>TRNAN-GUU | SCL/TAL1 interrupting locus<br>TRNAN-GUU | Microcephaly 7, primary, autosomal recessive | NM_003035.2:c.3751A>G<br>NM_001048166.1:c.3754A>G | NP_001041631.1:p.Ile1252Val<br>NP_003026.2:p.Ile1251Val | 3 | 0.0002 | rs142210835 | 0.0 | het. | 99.0 | 19 / 45 |
| 9 | 2635542 | VLDLR | very low density lipoprotein receptor | Cerebellar hypoplasia and mental retardation with or without quadrupedal locomotion 1 | NM_001018056.1:c.172_173del<br>NM_003383.3:c.172_173del | NP_001018066.1:p.Val59Ter<br>NP_003374.3:p.Val59Ter | 2 | 0.0001 | | 0.0 | het. | 99.0 | 23 / 37 |
| 1 | 74671377 | FPGT<br>FPGT-TNNI3K<br>TNNI3K<br>TRNAN-GUU | FPGT-TNNI3K readthrough<br>fucose-1-phosphate guanylyltransferase<br>TNNI3 interacting kinase<br>TRNAN-GUU | ?Cardiac conduction disease with or without dilated cardiomyopathy | NM_001112808.2:c.382+4283A>C<br>NM_001199328.2:c.923A>C<br>NM_001199329.2:c.*319A>C<br>NM_001199327.1:c.382+4283A>C<br>NM_003838.4:c.1685A>C | NP_001186257.2:p.Lys308Thr<br>NP_003829.3:p.Lys562Thr | 3 | 0.0002 | | 0.0 | het. | 99.0 | 20 / 46 |
| 1 | 53715098 | LRP8<br>TRNAN-GUU | low density lipoprotein receptor-related protein 8, apolipoprotein e receptor<br>TRNAN-GUU | {Myocardial infarction, susceptibility to} | NM_017522.4:c.2064+1264A>C<br>NM_004631.4:c.2807A>C<br>NM_033300.3:c.2297A>C<br>NM_001018054.2:c.2676+1264A>C | NP_150643.2:p.Gln766Pro<br>NP_004622.2:p.Gln936Pro | 3 | 0.0002 | | 0.0 | het. | 99.0 | 21 / 34 |
| 1 | 43893302 | SZT2<br>TRNAN-GUU | seizure threshold 2 homolog (mouse)<br>TRNAN-GUU | Epileptic encephalopathy, early infantile, 18 | NM_015284.3:c.3529C>T | NP_056099.3:p.Arg1177Trp | 3 | 0.0002 | | 0.0 | het. | 99.0 | 19 / 36 |
| 1 | 240371443 | FMN2 | formin 2 | Mental retardation, autosomal recessive 47 | NM_020066.4:c.3331_3363del | NP_064450.3:p.Pro1112_Pro1122del | 3 | 0.0005 | | 0.0 | het. | 87.0 | 3 / 4 |
| 1 | 240370975 | FMN2 | formin 2 | Mental retardation, autosomal recessive 47 | NM_020066.4:c.2863C>G | NP_064450.3:p.Pro955Ala | 3 | 0.0000 | | 0.0 | het. | 59.0 | 8 / 23 |
| 22 | 50660214 | TUBGCP6 | tubulin, gamma complex associated protein 6 | Microcephaly and chorioretinopathy, autosomal recessive, 1 | NM_020461.3:c.2574G>C | NP_065194.2:p.Trp858Cys | 3 | 0.0005 | rs147989796 | 0.004591 | hom. | 99.0 | 40 / 40 |
| 1 | 110884168 | RBM15<br>TRNAN-GUU | RNA binding motif protein 15<br>TRNAN-GUU | Megakaryoblastic leukemia, acute | NM_001201545.1:c.2141G>T<br>NM_022768.4:c.2141G>T | NP_001188474.1:p.Gly714Val<br>NP_073605.4:p.Gly714Val | 3 | 0.0007 | rs182719265 | 4.591E-4 | het. | 99.0 | 17 / 38 |
| 1 | 32669882 | CCDC28B<br>TRNAN-GUU | coiled-coil domain containing 28B<br>TRNAN-GUU | {Bardet-Biedl syndrome 1, modifier of} | NM_024296.3:c.427G>A | NP_077272.2:p.Glu143Lys | 3 | 0.0002 | | 0.0 | het. | 99.0 | 17 / 31 |
| 1 | 53793511 | LRP8<br>TRNAN-GUU | low density lipoprotein receptor-related protein 8, apolipoprotein e receptor<br>TRNAN-GUU | {Myocardial infarction, susceptibility to} | NM_033300.3:c.71_73dupTGC<br>NM_017522.4:c.71_73dupTGC<br>NM_001018054.2:c.71_73dupTGC<br>NM_004631.4:c.71_73dupTGC | NP_004622.2:p.Leu24dup<br>NP_150643.2:p.Leu24dup<br>NP_059992.3:p.Leu24dup<br>NP_001018064.1:p.Leu24dup | 3 | 0.0009 | | 0.0 | het. | 99.0 | 19 / 29 |
| 1 | 44071942 | PTPRF<br>TRNAN-GUU | protein tyrosine phosphatase, receptor type, F<br>TRNAN-GUU | ?Breasts and/or nipples, aplasia or hypoplasia of, 2 | NM_130440.2:c.3488_3490del<br>NM_002840.3:c.3515_3517del | NP_002831.2:p.Gln1173del<br>NP_569707.2:p.Gln1164del | 3 | 0.0002 | | 0.0 | het. | 99.0 | 12 / 24 |
| 2 | 179440424 | TTN<br>TTN-AS1 | titin<br>TTN antisense RNA 1 | Cardiomyopathy, familial hypertrophic, 9; Cardiomyopathy, dilated, 1G; Tibial muscular dystrophy, tardive; Muscular dystrophy, limb-girdle, type 2J; Myopathy, proximal, with early respiratory muscle involvement; Myopathy, early-onset, with fatal cardiomyopathy | NM_001267550.1:c.70435C>T<br>NM_001256850.1:c.65512C>T<br>NM_133432.3:c.43615C>T<br>NR_038272.1:n.2044-6875G>A<br>NM_003319.4:c.43240C>T<br>NM_133378.4:c.62731C>T<br>NM_133437.3:c.43816C>T<br>NR_038271.1:n.596+4248G>A | NP_597676.3:p.Arg14539Trp<br>NP_597681.3:p.Arg14606Trp<br>NP_596869.4:p.Arg20911Trp<br>NP_003310.4:p.Arg14414Trp<br>NP_001254479.1:p.Arg23479Trp<br>NP_001243779.1:p.Arg21838Trp | 3 | 0.0002 | | 0.0 | het. | 99.0 | 21 / 31 |
| [illegible] | [illegible] | [illegible] | [illegible] | [illegible] | [illegible] | [illegible] | [illegible] | [illegible] | [illegible] | [illegible] | [illegible] | [illegible] | [illegible] |

Page 1 of 1 - 1 to 26 of 26 variants Page size 1000
