# Supplementary material for: A 26-hour system of highly sensitive whole genome sequencing for emergency management of genetic diseases
Source: Genome Med. 2015 Sep 30;7:100. doi: 10.1186/s13073-015-0221-8 (PMC4588251; doi:10.1186/s13073-015-0221-8)
Supplement: Additional file 4: Table S1. — Comparison of the metrics of sequence yield and quality of 18-h and 26-h WGS (HiSeq 2500 2 × 100 nt rapid-run mode). a, R2 refers to read 2. Eighteen-hour runs had marginally better quality than 26-h runs, given slight differences in average cluster density. This might have been due to the shorter time of slide exposure to laser light and lesser loss in reagent stability. b. Comparison of 18-h and 26-h WGS metrics (HiSeq 2500 2 × 100 nt rapid-run mode), showing correlations between cluster density and metrics of sequence yield and quality. Cluster density explained much of the variability in yield, quality score, error rate, and % reads passing filter. (PDF 23 kb) [file 13073_2015_221_MOESM4_ESM.pdf]

a

Sample	Run Time (hr)	Sequence Yield (GB)	Total Reads	Cluster Density (K/mm²)	Nucleotides with Q score >30 (%)	Reads Passing Filter (%)	Raw Error rate (%)	Aligned Reads (%)	Aligned Reads with mapQ >20 (%)
CMH_184	26	137	1,539,534,606	1044	90	89	0.65	95.0	91.0
CMH_185	26	117	1,252,265,788	849	93	93	0.50	97.1	93.3
CMH_531	26	103	1,015,355,810	746	90.2	92.4	n.d.	97.8	94.1
CMH_569	26	101	995,793,286	1120	80.2	60.3	1.61	84.1	79.9
UDT_173	26	139	1,600,532,150	1085	89	87	0.55	94.7	90.6
	18	106	966,794,602	760	92.4	94	0.50	99.3	96.9
NA12878	18	143	1,330,334,428	1137	85	85	0.77	98.5	96.4
UDT_103	18	130	1,215,158,762	970	90	90.7	0.56	99.2	97.0

b

Metric	Sequence Yield (GB)	Cluster Density (K/mm²)	% > Q30	Passing Filter (%)	Error rate (%)
Correlation with cluster density	0.64		-0.72	-0.59	0.69
Mean of 18 Runs (n=3)	126.7	976.7	89.1	89.9	0.8
Mean of 26 Runs (n=5)	119.4	968.8	88.5	84.3	0.8
