# Supplementary figures and images for: A 26-hour system of highly sensitive whole genome sequencing for emergency management of genetic diseases
Source: Genome Med. 2015 Sep 30;7:100. doi: 10.1186/s13073-015-0221-8 (PMC4588251; doi:10.1186/s13073-015-0221-8)

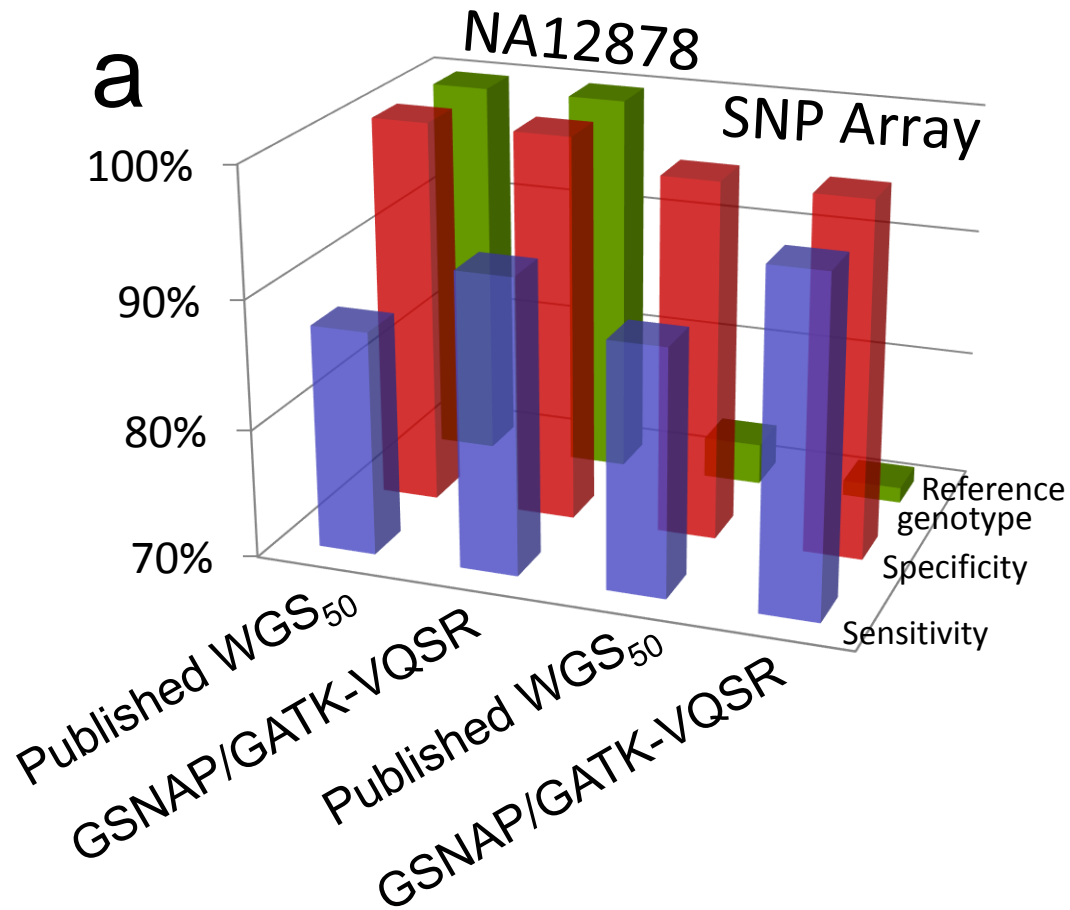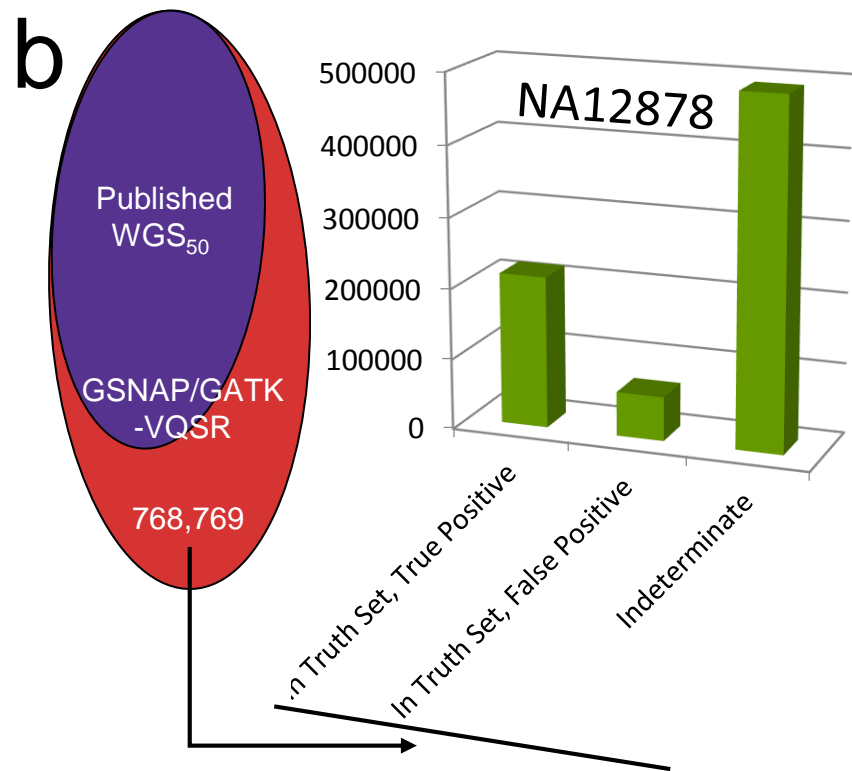

Supplement: Additional file 5: Figure S4. — Examination of the sensitivity and accuracy of nucleotide variant genotype calls in WGS with the published WGS50 and GSNAP/GATK-VQSR pipelines. A. Comparison of the sensitivity and accuracy of all nucleotide variant calls. B. Comparison of the accuracy of variants that were uniquely called by the GSNAP/GATK-VQSR. WGS was performed using the HiSeq 2500 with 2 × 100 cycles and 18-h run time. The sample UDT_173 genotype “truth set” was from hybridization to the Omni4 SNP array. The NA12878 ‘truth set’ was from ftp://ftp-trace.ncbi.nih.gov/giab/ftp/data/NA12878/variant_calls/NIST26. (PDF 213 kb) [file 13073_2015_221_MOESM5_ESM.pdf]
